# *α*-amino-3-hydroxy-5-methyl-4-isoxazole propionic acid (AMPA) receptor density underlies intraregional and interregional functional centrality

**DOI:** 10.3389/fncir.2024.1497897

**Published:** 2024-11-06

**Authors:** Taisuke Yatomi, Dardo Tomasi, Hideaki Tani, Shinichiro Nakajima, Sakiko Tsugawa, Nobuhiro Nagai, Teruki Koizumi, Waki Nakajima, Mai Hatano, Hiroyuki Uchida, Takuya Takahashi

**Affiliations:** ^1^Department of Neuropsychiatry, Keio University School of Medicine, Tokyo, Japan; ^2^Department of Physiology, Yokohama City University Graduate School of Medicine, Yokohama, Japan; ^3^Laboratory of Neuroimaging (LNI), National Institute on Alcohol Abuse and Alcoholism, NIH, Bethesda, MD, United States

**Keywords:** *α*-amino-3-hydroxy-5-methyl-4-isoxazole propionic acid (AMPA) receptor, [^11^C]K-2, positron emission tomography, synaptic plasticity, resting-state functional MRI (rsfMRI), functional connectivity density mapping, functional network, functional centrality

## Abstract

Local and global functional connectivity densities (lFCD and gFCD, respectively), derived from functional magnetic resonance imaging (fMRI) data, represent the degree of functional centrality within local and global brain networks. While these methods are well-established for mapping brain connectivity, the molecular and synaptic foundations of these connectivity patterns remain unclear. Glutamate, the principal excitatory neurotransmitter in the brain, plays a key role in these processes. Among its receptors, the *α*-amino-3-hydroxy-5-methyl-4-isoxazole propionic acid receptor (AMPAR) is crucial for neurotransmission, particularly in cognitive functions such as learning and memory. This study aimed to examine the association of the AMPAR density and FCD metrics of intraregional and interregional functional centrality. Using [^11^C]K-2, a positron emission tomography (PET) tracer specific for AMPARs, we measured AMPAR density in the brains of 35 healthy participants. Our findings revealed a strong positive correlation between AMPAR density and both lFCD and gFCD-lFCD across the entire brain. This correlation was especially notable in key regions such as the anterior cingulate cortex, posterior cingulate cortex, pre-subgenual frontal cortex, Default Mode Network, and Visual Network. These results highlight that postsynaptic AMPARs significantly contribute to both local and global functional connectivity in the brain, particularly in network hub regions. This study provides valuable insights into the molecular and synaptic underpinnings of brain functional connectomes.

## Introduction

Functional magnetic resonance imaging (fMRI) is widely used to examine the functional connectivity between different areas and functional networks in the human brain using correlation analysis (Logothetis, 2008). However, the molecular and synaptic basis of functional connectivity remains unclear. Hypothesis-driven seed correlation analysis is advantageous when there is strong theoretical support for extracting regions of interest (ROIs) (Fox and Raichle, 2007). Alternatively, data-driven functional connectivity density (FCD) metrics map hubness (Tomasi and Volkow, 2010), the number of functional connectivities with all other brain voxels, without needing predefined ROIs (Zhang and Volkow, 2019).

Prior studies have shown that higher functional connectivity density is supported by higher metabolic rate of glucose (Tomasi et al., 2013; Shokri-Kojori et al., 2019). Indeed, strong correlations have been found between glucose metabolism and FCD in the default mode network (DMN), dorsal attention network (DAN), and visual network (VN) (Tomasi et al., 2013). However, glucose metabolism is an indirect indicator of neural activity. Alteration of functional connectivity density could be seen in several neurological and psychiatric disorders such as Parkinson’s disease (Hu et al., 2017), epilepsy (Li et al., 2019), cognitive impairment (Miao et al., 2022; Song et al., 2022), substance-use disorders (Konova et al., 2015; Manza et al., 2018), developmental disorders (Tomasi and Volkow, 2012; Tomasi and Volkow, 2019), schizophrenia (Li et al., 2022), and depression (Zhang et al., 2016; Zou et al., 2016). Nonetheless, no studies have examined the effect of neural activity at a synaptic level on functional connectivity in living humans.

Molecular imaging has become increasingly important for a comprehensive understanding of the human functional connectome. Molecular imaging can provide insights into neurotransmission in chemical synapses, which imaging modalities like MRI and electrophysiology cannot (Sala et al., 2023). Recently, efforts have been made to generate brain atlases of various synapses, with the aim of applying these to translational research (Zachlod et al., 2023). The glutamatergic nervous system is hypothesized to be the basis of brain function (Reiner and Levitz, 2018) and is implicated in psychiatric illnesses such as schizophrenia (Uno and Coyle, 2019) and mood disorders (Guglielmo et al., 2022). Glutamate is a pivotal neurotransmitter responsible for excitatory neurotransmission in the brain, and glutamatergic synapses account for the vast majority of all excitatory synapses in the brain (Nakanishi, 1992; Traynelis et al., 2010). Among the glutamate receptors, *α*-amino-3-hydroxy-5-methyl-4-isoxazole propionic acid receptor (AMPAR) plays a crucial role in conveying fast excitatory synaptic transmission and underlies synaptic plasticity (Malinow and Malenka, 2002), which is the fundamental of learning and memory (Diering and Huganir, 2018). The synaptic trafficking of AMPARs is a crucial molecular mechanism underlying experience-dependent synaptic plasticity (Takahashi et al., 2003; Kessels and Malinow, 2009; Mitsushima et al., 2011; Mitsushima et al., 2013; Miyazaki et al., 2021). Furthermore, previous reports showed that AMPAR had interactions with other neurotransmitters such as striatal-enriched protein tyrosine phosphatase 61, which also regulated N-methyl-D-aspartate (Won et al., 2019), and with a variety of proteins that contribute to its complex regulation (Matthews et al., 2021). AMPAR is protected from desensitization during synaptic activity by Shisa6, a single transmembrane protein and a stable and directly interacting AMPAR auxiliary subunit (Klaassen et al., 2016), which supports our hypothesis that AMPARs centrally comprise functional interactomes via many types of proteins and neurotransmitters. Experience-dependent synaptic reorganization can underlie the functional connectome, and thus, AMPAR can be a key synaptic molecule supporting the functional networks in the brain. We have recently developed the positron emission tomography (PET) for AMPAR, [^11^C]K-2, the first technology to visualize and quantify AMPAR (Miyazaki et al., 2020). [^11^C]K-2 depicts AMPAR on the cell surface which is a physiologically crucial fraction (Arisawa et al., 2021).

Using the ligand [^11^C]K-2, this study aimed to examine the association between AMPAR density and functional connectivity. We used three data-driven FCD metrics (Tomasi and Volkow, 2010) because there were no previous studies identifying specific functional connectivity pathways related to AMPAR in humans: local FCD (lFCD) (i.e., short-range FCD) maps how many nearby voxels are significantly connected to every voxel in the brain, the number of edges at a certain voxel in a cluster, and indexes short-range (intraregional) centrality; global FCD (gFCD) maps the total number of voxels connected with every brain voxel; and the difference between gFCD and lFCD (i.e., long-range FCD) which indexes interregional functional centrality (Tomasi and Volkow, 2012).

Since AMPARs facilitate synaptic transmission and plasticity, processes essential for establishing and maintaining neural connections, we hypothesized a positive correlation between AMPAR density and short-and long-range FCD patterns across the whole brain and in each brain region or network.

## Materials and methods

### Ethics statement

The data for this study were derived from the studies approved by the Yokohama City University Human Investigation Committee and Yokohama City University Certified Institutional Review Board, following the ethical guidelines for medical and health research involving human participants by the Japan Ministry of Health, Labour and Welfare and the Clinical Trials Act in Japan (jRCTs031180052, jRCTs031200083). The study also conformed to the Declaration of Helsinki. All participants provided written informed consent after receiving detailed information about the protocol and demonstrated sufficient decision-making capacity as measured by the MacArthur Competence Assessment Tool for Clinical Research (Appelbaum and Grisso, 2001).

### Participants

We included 35 healthy participants from two studies. All participants were registered and recruited by the medical research recruitment company Cismor. Five of the participants were extracted from the first study (jRCTs031180052). This study examined the feasibility of [^11^C]K-2 PET imaging in living humans including healthy individuals and patients with psychiatric disorders. The participants were male, aged 30–49 years, capable of providing informed consent, and did not show any diagnostic criteria for psychiatric conditions according to the Diagnostic and Statistical Manual of Mental Disorders Fourth Edition (DSM-IV) (American Psychiatric Association, 1994) criteria using the structured clinical interview for DSM-IV (First et al., 1996), the DSM Fifth Edition (American Psychiatric Association, 2013) criteria, and the International Classification of Diseases Tenth Edition (World Health Organization, 1992) criteria. The remaining 30 healthy participants were included from a second study (jRCTs031200083). This study included participants with broader ranges of age and sex and examined differences in AMPAR distribution between healthy individuals and patients with psychiatric disorders, such as depression, bipolar disorder, schizophrenia, and autism spectrum disorder. It was conducted to determine biological differences in AMPAR between patients with psychiatric disorders and healthy individuals. The inclusion criteria for the second study were the same as those in the first study, except for the age range (20–69 years) and sex (males and females). The exclusion criteria were the same for the two studies; individuals were excluded if they were pregnant, nursing, or wishing to be pregnant, had a history of epilepsy, met criteria for substance abuse within six months before the study; had a positive urine drug screen for illicit drugs; received treatment with perampanel; met contraindications for MRI scan; had a significant neurological or general medical condition; or showed abnormal laboratory test values of serum creatinine ≥1.5 mg/dL, aspartate aminotransferase ≥150 IU/L, or alanine aminotransferase ≥150 IU/L. We used single-center data to ensure homogeneity of imaging data in the current study, and the age of the original participants in the data used in this study ranged from 24 to 49 years.

### *In vivo* PET imaging

The participants underwent PET with [^11^C]K-2 and MRI. PET imaging was performed using a TOSHIBA Aquiduo scanner (TOSHIBA Medical) and a Celesteion PCA-9000A/2A scanner (Canon Medical). These two types of PET had to be used for administrative and operational reasons in parallel. Aquiduo provided an axial FOV of 240 mm and 80 contiguous 2.0 mm thick slices. A 4.7 s transmission scan was performed for attenuation correction (AC), then a 60 s intravenous injection of [^11^C]K-2 (372.9 ± 14.1 MBq) was given with the flow rate of 60 ~ 80 μL/s, which was followed by an emission scan of 60 min in all studies, with frames of 18 × 10 s, 2 × 30 s, 7 × 60 s, 1 × 2 min, 1 × 3 min, 3 × 5 min and 3 × 10 min. Dynamic images were reconstructed with a 2D-Ordered Subset Expectation Maximization (OSEM) using four iterations, 14 subsets, a 128 matrix, a zoom with Gaussian kernel of 2.8 and 5.0 mm full width at half maximum (FWHM). Celesteion provided an axial FOV of 240 mm and 96 contiguous 2.0 mm thick slices. A 15.2 s transmission scan was performed for AC, and a 60 s intravenous injection of [^11^C]K-2 (376.8 ± 8.1MBq) was administered with the flow rate of 60 ~ 80 μL/s, followed by an emission scan of 60 min, with 35 frames. Dynamic images were reconstructed with 3D-OSEM coupled with Time of Flight (TOF) using two iterations, 20 subsets, a 128 matrix, a zoom with Gaussian kernel of 1.0 and 5.0 mm FWHM. Sixteen of all thirty-five healthy participants were allocated to the Aquiduo scanner, and nineteen of all participants were scanned with the Celesteion scanner.

### PET camera validation using phantom

To determine reconstruction parameters of PET images, and validate PET cameras, we performed PET scans on all types of PET cameras using brain tumor (BT) phantom (Itoi Factory Inc.) 13, which has multiple spheres of different sizes (diameter 7.5, 10, 13, 16, 27, 38mm) placed inside the cavity. The background area and the spheres of the BT phantom were filled with the activity of 5 and 10 kBq/mL using 18F-FDG, respectively. The evaluation was made based on three criteria: (1) recovery rate, (2) uniformity, and (3) quantitative performance. How to set up a circular ROI for recovery analysis: we performed this on the slice in which the sphere of each size was most clearly depicted. Then, we set an ROI with the same size as each sphere and 10 circular ROIs with a size of about 100 mm2 in the background area (6 pieces at a distance of 15 mm or more from the phantom edge and 4 pieces at the center). How to set ROI for uniformity analysis: We performed this for three slices in which no sphere is depicted. Then we used slices separated by at least 10 mm from each other. We set 16 circular ROIs with a size of about 100 mm2 in the background area (12 at a distance of 15 mm or more from the phantom edge and 4 at the center). For each ROI, we calculated the average value of SUV (SUVmean). The evaluation criteria were defined as follows. 1) recovery rate: SUVmean of each HOT sphere is 90% or more for 38mm sphere, 85% or more for 27mm sphere, 70% or more for 20mm sphere, and 60% or more with 16mm sphere. 2) Uniformity: The standard deviation of the relative error of SUVmean for the set ROI was 0.0249 or less. (C) Quantitative performance: the SUVmean of the set ROI should fall within the range of 0.95 to 1.05 against the theoretical value. We confirmed that all PET cameras used in this study could meet these criteria.

### MRI acquisition

Each participant underwent an MRI scan on a GE DISCOVERY MR750 3.0 T with 3.0T GEM Coil Suite (General Electric Medical Systems) to permit accurate delineation of the brain regions for data analysis. High-resolution 3D-T1-weighted images (T1WI) were acquired using the following parameters with the sequence of 3D BRAVO: voxel size = 0.9 × 0.9 × 0.9 mm, repetition time (TR) /time to echo (TE) = 7.0/3.1 ms, flip angle (FA) = 8°, FOV =  230 mm × 230 mm × 180 mm, and Matrix = 256 × 256 × 200. Resting-state functional MRI (rsfMRI) data were acquired with a T2*-weighted gradient-echo echo planar imaging sequence and the following parameters: TR, 2500 ms; TE, 30 ms; flip angle (FA) = 80°, matrix, 64 × 64; slice thickness, 3.2 mm; voxel size, 3.3 × 3.3 × (slice thickness) 3.2 mm^3^; the number of time points, 240; and scanning duration, 10 min. A 70 cm inner diameter built-in body coil was used for RF transmission, and a Head Neck Unit in a 19-channel (12 channels of them were used at a head scan) GEM Coil 3.0T (49.5 cm × 38.8 cm × 35.4 cm) was used for reception.

### MRI data preprocessing

Structural and functional images were preprocessed with the fMRIPrep v23.2.0 (Esteban et al., 2019), which is based on Nipype v1.8.6 (Gorgolewski et al., 2011). This process used tools from FMRIB Software Library (FSL) v6.0.3 (Jenkinson et al., 2012), Analysis of Functional NeuroImages (AFNI) v22.0.11 (Cox, 1996; Cox and Hyde, 1997), Advanced Normalization Tools (ANTs)^1^ v2.3.5, and Freesurfer^2^ v7.1.1. Functional images processed after fMRIPrep were denoised with XCP-D v0.6.0 (Ciric et al., 2018; Mehta et al., 2024). These pipelines were conducted as follows: (1) intensity non-uniformity correction with N4 bias correction which applied N4 algorithm implemented in ANTs and skull-stripping for T1WI; (2) T1 segmentation into gray matter (GM), white matter (WM), and cerebrospinal fluid (CSF); (3) slice timing correction; (4) distortion-correction with participant-specific fieldmap images; (5) realignment of all volumes to a selected reference volume including the estimation of head motion; (6) normalization to the Montreal Neurological Institute (MNI) space with the ICBM 152 Nonlinear Asymmetrical template version 2009c (Fonov et al., 2011; Manera et al., 2020); (7) surface driven alignment of functional and anatomical MRI data; (8) censoring of high-motion outlier, framewise displacement calculation, and thresholding; (9) bandpass filtering with a passband between 0.01–0.08 Hz; and (10) confound regression with six motion estimates, WM, CSF, and global signal. Normalized fMRI time series were resampled to a 2 mm isotropic voxel size. To take advantage of the most probable area that has WM, we segmented WM independently of the segmentation in fMRIPrep to establish a reference region. The criteria for segmentation included a probability of the presence of WM > 0.9, the 8 mm-smoothed GM < 0.05, and the 8 mm-smoothed CSF < 0.05, with Statistical Parametric Mapping 8 (SPM 8) (Miyazaki et al., 2020).

### AMPAR-PET preprocessing

A summed image 30–50 min after the injection of [^11^C]K-2 was obtained for all PET images of each participant using the PMOD PNEURO tool v3.8 (PMOD Technologies) as 30–50 min is the best time frame for the quantitative analysis (Miyazaki et al., 2020). Standardized uptake value ratio (SUVR)_30–50 min_ images were obtained by dividing the radioactivity values by a reference region of WM. The volume of interest of the WM was obtained by fulfilling the following conditions for voxel value: probability of the presence of WM > 0.9, the 8 mm-smoothed GM < 0.05, and the 8 mm-smoothed CSF < 0.05, using SPM 8 (Miyazaki et al., 2020). Because we found a good linear relationship between (SUVR)_30–50 min_ and a non-displaceable binding potential (BP_nd_), which is a quantitative index of receptor density and is commonly utilized, in the previous study (Miyazaki et al., 2020), we used (SUVR)_30–50 m_ as the representative of AMPAR density. (SUVR)_30–50 min_ images were processed as follows. SUVR images were co-registered to respective original T1WI images and normalized with the transformation information from native T1WI to the ICBM 152 Nonlinear Asymmetrical template version 2009c (Fonov et al., 2011; Manera et al., 2020) to the template using ANTs. The preprocessed SUVR images were then smoothed with a Gaussian kernel of 8 mm full width at half maximum (FWHM). Subsequently, SUVR images were restricted to areas of SUVR >1 using AFNI because we targeted areas to be analyzed in areas where AMPAR and WM had almost no AMPAR, according to a previous report (Miyazaki et al., 2020). To restrict SUVR images to GM, we used a 2 mm isotropic-resampled GM mask. This mask, which only includes areas with image values above 10%, was extracted from standard tissue probability maps available in SPM12 (Wellcome Trust Centre for Neuroimaging, London, UK; https://www.fil.ion.ucl.ac.uk/spm/) in MATLAB R2019b. Normalized SUVR images were resampled to a 2 mm isotropic voxel size.

### Functional connectivity density mapping

Pearson’s correlation was calculated to assess the strength of functional connectivity, C_ij_, between voxels i and j. We defined a local functional connectivity graph G = (V, E), where each brain voxel is represented as a vertex (V) in the graph. An edge (E) is drawn between two voxels, v_i_ and v_j_, if the temporal correlation of their fMRI signals C_ij_ exceeds 0.6 (Tomasi and Volkow, 2010; Thompson et al., 2016) and v_j_ is part of a spatially connected cluster of voxels that includes v_i_, and lFCD was defined as the number of edges associated with G_i_. In analogy to G, we also defined global functional connectivity graph H = (W, F), where each brain voxel is represented as a vertex (W). An edge (F) connects two vertices, w_i_ and w_j_ if the temporal correlation of their fMRI signals C_ij_ exceeds 0.6, regardless of whether w_j_ is part of a spatially connected cluster with w_i_. Additionally, gFCD was defined as the number of edges associated with H_i_. Since the number of voxels that gFCD estimated included the number of voxels estimated by lFCD, gFCD-lFCD was used as the interregional functional centrality. The lFCD algorithm (Tomasi and Volkow, 2010) calculates whether an original voxel is significantly connected to other voxels and radially adjacent to each other (including voxels that are not necessarily adjacent to the original voxel), and stops the calculation when there are no more adjacent significantly connected voxels around the cluster formed with the original voxel. If we consider a cluster around the original voxel created in this way as a “region” connected to each other by significant connectivity, we can define lFCD as intraregional functional centrality and then voxels and clusters that are not connected to that region but have significant connectivity with the original voxel will be contained in a different “region” than the “region” containing the original voxel. Thus, we can define gFCD-lFCD, which represents the number of connectivities between the original voxel and those distant regions, as the functional centrality between regions, named as interregional functional centrality. To improve the normal distributions of lFCD and gFCD, we used log (lFCD) [i.e., short-range FCD (srFCD)] and log (gFCD-lFCD) [i.e., long-range FCD (lrFCD)]. We used the terms “srFCD” and “lrFCD” differentiated from lFCD and gFCD-lFCD, respectively, for two reasons. First of all, they included the literally critical meanings that we wanted to express, srFCD as functional connectivity density within a short range and lrFCD as functional connectivity density within a long range. In addition, they have been used in the previous reports and we thought that the use of their terms was useful for interpretation. An isotropic 8 mm FWHM Gaussian kernel was used for the spatial smoothing of the srFCD and lrFCD images. FCD calculations were carried out in AFNI using 3dLFCD and 3dDegreeCentrality functions (Craddock and Clark, 2016). In this study, functional centrality indicates Functional Connectivity Density (FCD), whose meaning is based on the number of significant functional connectivity with other voxels in each original voxel. Functional centrality is originally derived from the meaning of “Degree Centrality,” which is a concept of graph theory in a broader sense. In other words, “Degree Centrality” is the degree of centrality of how many voxels a voxel is central to a connected voxel, and functional centrality is a definition of the term that includes the definition (Telesford et al., 2011). In the whole parts, we used the terms srFCD and lrFCD, where we only want to describe the facts of the results. On the other hand, we used the term functional centrality when we want to emphasize the concept “Degree Centrality” with a view to generalizing our results and discussions.

### Statistical analyses

The mean of 35 preprocessed SUVR images was calculated to examine the relationship between AMPAR and srFCD or lrFCD across the entire brain. Similarly, the mean of 35 srFCD or 35 lrFCD images were calculated. Within each ROI delineated by the Hammers atlas (Hammers et al., 2003; Gousias et al., 2012), as well as all seven networks defined in Yeo’s 7-network atlas (Yeo’s atlas) (Thomas Yeo et al., 2011), we calculated mean values of SUVR, srFCD, and lrFCD. After we confirmed the distribution patterns of SUVR, srFCD, lrFCD, and gFCD did not conform to normal distribution with the Kolmogorov–Smirnov test (Supplementary Table S6), the Spearman rank correlation test was used to ascertain whether a statistically significant correlation existed between SUVR and srFCD or lrFCD across the 83 ROIs of the Hammers Atlas. The Spearman rank correlation test was also used to assess the voxel-wise correlation between SUVR and srFCD or lrFCD within each ROI or network for each participant. Subsequently, Fisher’s z-transformation was applied to normalize the step-distributed correlation coefficients. We performed multiple comparison correction in the following two steps. Step 1: The Bonferroni correction was used to correct for multiple comparisons for 83 ROIs and 7 networks. Thus, statistical significance was defined by *p* < 0.05/90. R version 4.1.3 (R Core Team, 2022) was used for this purpose. Step 2: spin-based permutation correction was conducted to control potential spatial autocorrelation to the whole brain correlation analysis and to the voxel-based analyses in ROIs in Hammers atlas (correlation between SUVR and srFCD: 33 areas survived after step1, correlation between SUVR and lrFCD: 41 areas survived after step1) and 7 areas in Yeo’s atlas after step 1. In step 2, the mean SUVR, srFCD, and lrFCD images were resampled to 1 mm^3^ spatial resolution to warrant to extract values from the voxel of the precise coordinates of vertices in the normalized surface template space in Freesurfer applied in the spin-test (Alexander-Bloch et al., 2018). Those image values in the coordinates of 10242 vertices for the bilateral hemisphere were extracted and projected onto the normalized sphere data implemented in Freesurfer fsaverage5 (see text footnote 2). All sphere data were rotated at angles uniformly chosen between zero and 360, about each of the x (left–right), y (anterior–posterior), and z (superior–inferior) axes. Each permutation-rotated SUVR was tested for correlation with srFCD or lrFCD, and the correlation coefficients were stored for comparison with the correlation between the original SUVR and srFCD or lrFCD. The testing probability was defined as the number of the null correlations (the results of correlation using 1000 times permutation) outperforming the real correlation being divided by 1000 in each ROI or network, and statistical significance was set to *p* < 0.05 in step 2. The subcortex areas and bilateral cerebellum areas were excluded in step 2 (in correlations between SUVR and srFCD: the bilateral cerebellum and right putamen, in correlations between SUVR and lrFCD: the bilateral cerebellum, putamen, and caudate) because the spin-test does not cover those areas for permutation (Alexander-Bloch et al., 2018). Step 2 results are shown in Supplementary Tables S3, S4. To assess the correlation between absolute values of FCD and the degree of the correlation between SUVR and FCD, we performed the Spearman’s rank correlation test after the above 2 steps correction to calculate the correlation between srFCD values and correlation coefficients between SUVR and srFCD using the corrected 32 regions (including the bilateral cerebellum and right putamen) and the correlation between lrFCD values and correlation coefficients between SUVR and lrFCD using the corrected 41 regions (including the bilateral cerebellum, putamen, and caudate), where *p*-value <0.05 was recognized as significant. Before those calculations, we also confirmed that the distribution patterns of srFCD and lrFCD values in Hammers ROIs and correlations between SUVR and srFCD or lrFCD were not normal, respectively, with the Shapiro–Wilk test (Supplementary Table S7). Normality was not met in each data except for correlations (z values) between SUVR and srFCD. We also calculated correlations between SUVR and gFCD in 83 ROIs in Hammers Atlas, 7 networks in Yeo’s Atlas according to the same manner in the above methods also using the above 2 steps’ correction (Supplementary Figure S1; Supplementary Table S5). The subcortex areas (the bilateral cerebellum, putamen, and caudate) and bilateral cerebellum areas were also excluded in calculating correlations between SUVR and gFCD in step 2 because the spin-test does not cover those areas for permutation (Alexander-Bloch et al., 2018). Step 2 was done with python version 3.9.7 (extraction of vertex values in SUVR, srFCD, lrFCD, and gFCD images), Matlab R2019b (to conduct spin-test), and R version 4.1.3 (for calculation of images such as multiplication and addition between images).

## Results

We conducted a PET scan with [^11^C]K-2 and a resting-state fMRI (rsfMRI) scan in thirty-five healthy participants aged 38.1 ± 7.1 years (mean ± SD), including 22 males (62.9%).

### ROI-wise positive correlation among the whole brain analysis

**Figure 1:**
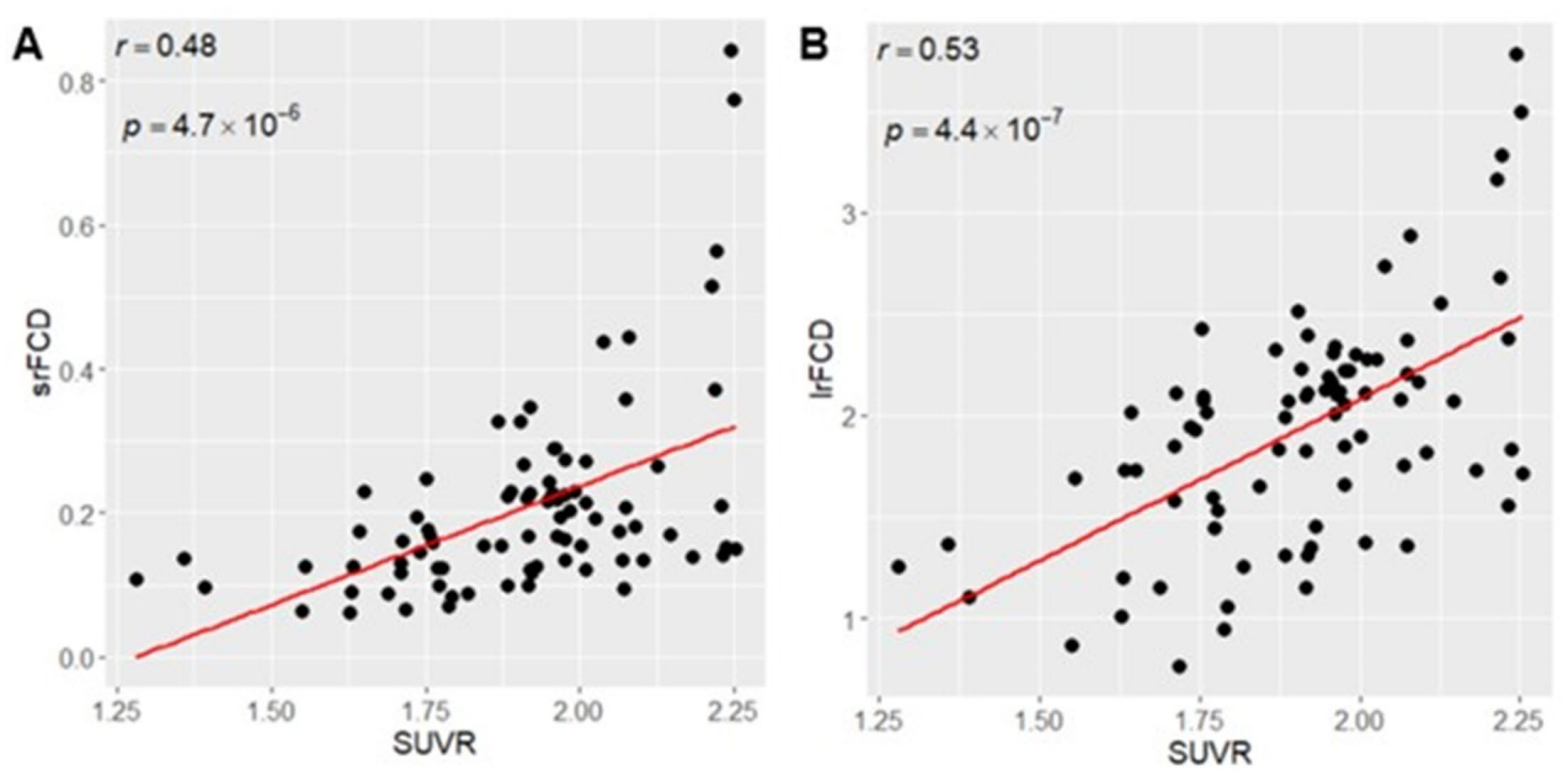
(A) Positive Correlation between SUVR mean and srFCD mean. (B) Positive correlation between SUVR mean and lrFCD mean. Each dot indicates each ROI in Hammers atlas. srFCD, short-range functional connectivity density; SUVR, standardized uptake value ratio; ROI, region of interest; lrFCD, long-range functional connectivity density.

To investigate the relationship between SUVR and FCD across the whole brain, we calculated mean SUVR, srFCD, and lrFCD images across all participants. We calculated the average values of these mean images within 83 ROIs in the Hammers atlas and tested the correlation between mean SUVR and mean srFCD or mean lrFCD across ROIs. We found a significant positive correlation between SUVR and srFCD (*r* = 0.48, *p* = 4.7 × 10^−6^; Figure 1A) and between SUVR and lrFCD (*r* = 0.53, *p* = 4.4 × 10^−7^; Figure 1B). These results suggest a positive correlation between increased SUVR and increased intraregional and interregional functional centralities in humans. To account for potential spatial autocorrelation, we conducted a spin-based permutation test (Alexander-Bloch et al., 2018) to validate the correlation between SUVR and srFCD or lrFCD across the entire cortex. We projected the mean SUVR for each of the 20,484 vertices across the left and right hemispheres (10,242 vertices per hemisphere) onto normalized spherical data using Freesurfer. The spherical coordinates were then rotated 1,000 times at random angles (0–360 degrees) around the x (left–right), y (anterior–posterior), and z (superior–inferior) axes, as per the spin-test methodology (Alexander-Bloch et al., 2018). We compared the real correlation coefficients (0.53 for srFCD and 0.63 for lrFCD) with those from the null distribution generated by the spin-test. In both cases, the null hypothesis was rejected (*p* < 0.001), confirming the robustness of the observed correlations.

### Voxel-wise regional analysis within anatomical ROIs

**Figure 2:**
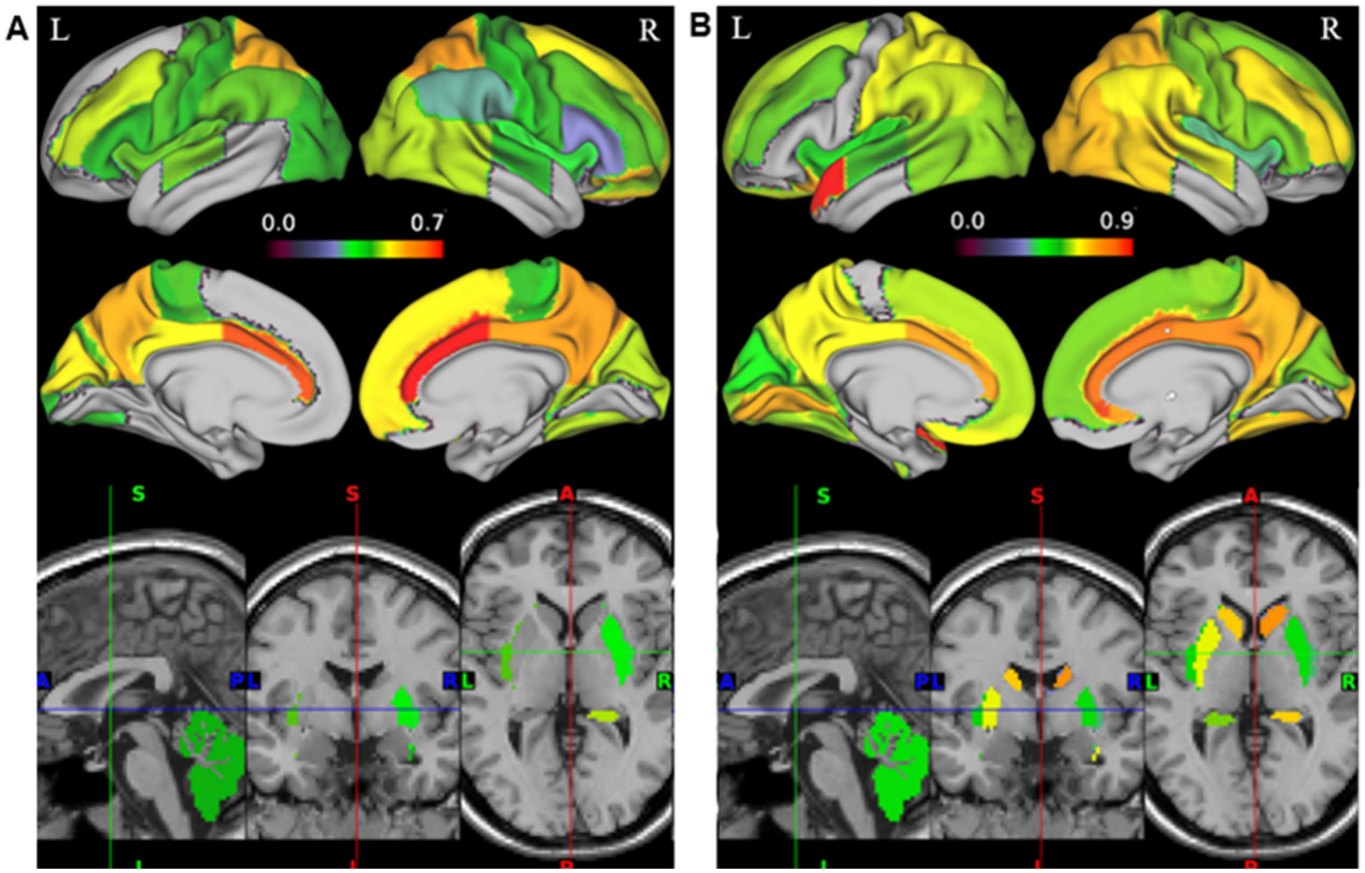
Correlation map between AMPAR density (SUVR) and FCD in 35 healthy participants. Color gradation of each ROI in Hammers atlas reflects the degree of the mean correlation coefficient. (A) Correlation map between SUVR and srFCD. 32 regions where all the participants showed adjustments were displayed (*p* < 0.05/90). All 29 regions in the cortex were spatial autocorrelation-corrected with spin-based permutation (*p* < 0.05). (B) Correlation map between SUVR and lrFCD. 41 regions where all the participants showed statistically significant correlation coefficients after multiple comparison adjustments were displayed (*p* < 0.05/90). All 36 regions in the cortex were spatial autocorrelation-corrected with spin-based permutation (*p* < 0.05). AMPAR, *α*-amino-3-hydroxy-5-methyl-4-isoxazole propionic acid receptor; lrFCD, long-range functional connectivity density; srFCD, short-range functional connectivity density; SUVR, standardized uptake value ratio; FCD, functional connectivity density.

**Figure 3:**
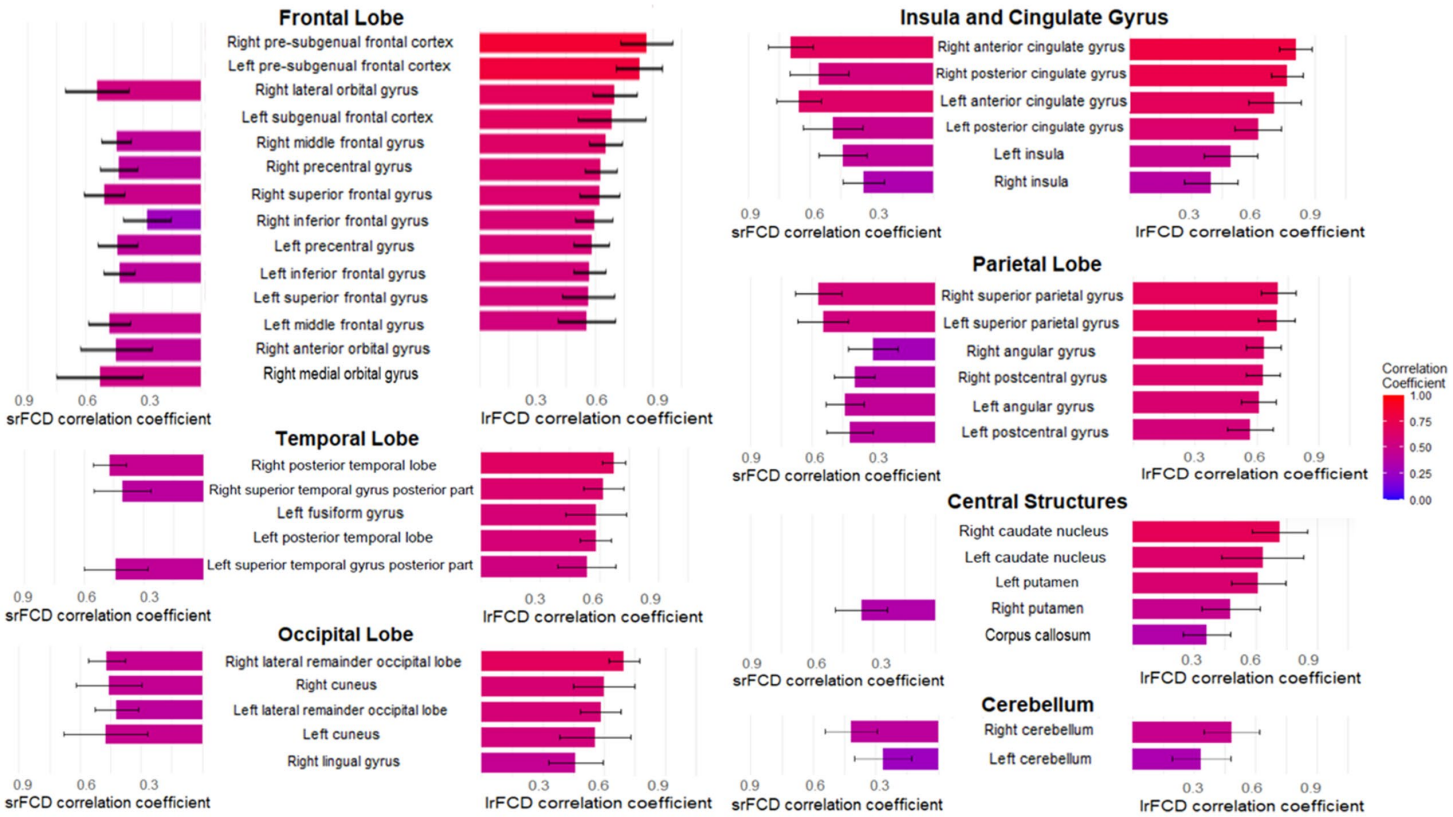
The mean correlation coefficients of multiple comparison-corrected and spatial autocorrelation-corrected areas in Hammers atlas between AMPAR density (SUVR) and FCD in 35 healthy participants. The bar graphs in the left column shows the mean correlation coefficients between SUVR and srFCD. The bar graphs in the right column shows the mean correlation coefficients between SUVR and lrFCD. The names in the center at the same height as these bars are those of the corresponding brain regions in Hammers Atlas. The large headings follow the major divisions of Hammers Atlas. The mean correlation coefficients of 32 areas between SUVR and srFCD are corresponding to Figure 2A. The mean correlation coefficients of 41 areas between AMPAR density (SUVR) and lrFCD are corresponding to Figure 2B. Bars represent the mean and whiskers show the SD. AMPAR, α-amino-3-hydroxy-5-methyl-4-isoxazole propionic acid receptor; lrFCD, long-range functional connectivity density; srFCD, short-range functional connectivity density; SUVR, standardized uptake value ratio; FCD, functional connectivity density; SD, standard deviation.

We tested for differences in the relationship between SUVR and FCD within 83 ROIs. Only positive correlations between SUVR and srFCD or lrFCD were statistically significant within ROIs. The highest mean correlations between SUVR and srFCD were observed in the bilateral anterior cingulate cortex (ACC) (left: *r* = 0.67, right: *r* = 0.71), bilateral posterior cingulate cortex (PCC) (left: *r* = 0.50, right: *r* = 0.57), and bilateral superior parietal gyrus (left: *r* = 0.56, right: *r* = 0.58) (Figures 2A, 3). Conversely, regions with low mean correlations (*r* < 0.30) (Cohen, 1992) between SUVR and srFCD included the left cerebellum (*r* = 0.27), right inferior frontal gyrus (*r* = 0.28) (Figures 2A, 3). For the correlation between SUVR and lrFCD, the highest mean correlations were found in the bilateral ACC (left: *r* = 0.73, right: *r* = 0.82), bilateral PCC (left: *r* = 0.64, right: *r* = 0.78), bilateral caudate nucleus (left: *r* = 0.68, right: *r* = 0.74), left subgenual frontal cortex (left: *r* = 0.73), and bilateral pre-subgenual frontal cortex (psFC) (left: *r* = 0.86, right: *r* = 0.91) (Figures 2B, 3B). None of the regions exhibited low mean correlation coefficient (r < 0.30) between SUVR and lrFCD. We further applied spatial autocorrelation correction using the spin-test (Alexander-Bloch et al., 2018) on ROIs that survived multiple comparison corrections—35 ROIs for the correlation between SUVR and srFCD, and 46 ROIs for the correlation between SUVR and lrFCD. Initially, we identified which vertices in the normalized surface data (totaling 20,484 vertices across the entire brain) corresponded to each ROI in the Hammers atlas. Using the spherical mean SUVR data calculated for all vertices, we assessed whether the correlation between mean SUVR and mean srFCD or lrFCD within each ROI was greater than the correlations obtained from 1,000 spin-based permutations of SUVR in each multiple comparison-corrected ROI. After correcting for spatial autocorrelation in the cortex, we identified 29 ROIs with significant correlations between SUVR and srFCD, and 35 ROIs with significant correlations between SUVR and lrFCD (Figures 2A,B; Supplementary Tables S3, S4). These results suggest that certain brain areas facilitate communication between AMPAR-related excitatory signals and functional centrality more efficiently than others.

**Figure 4:**
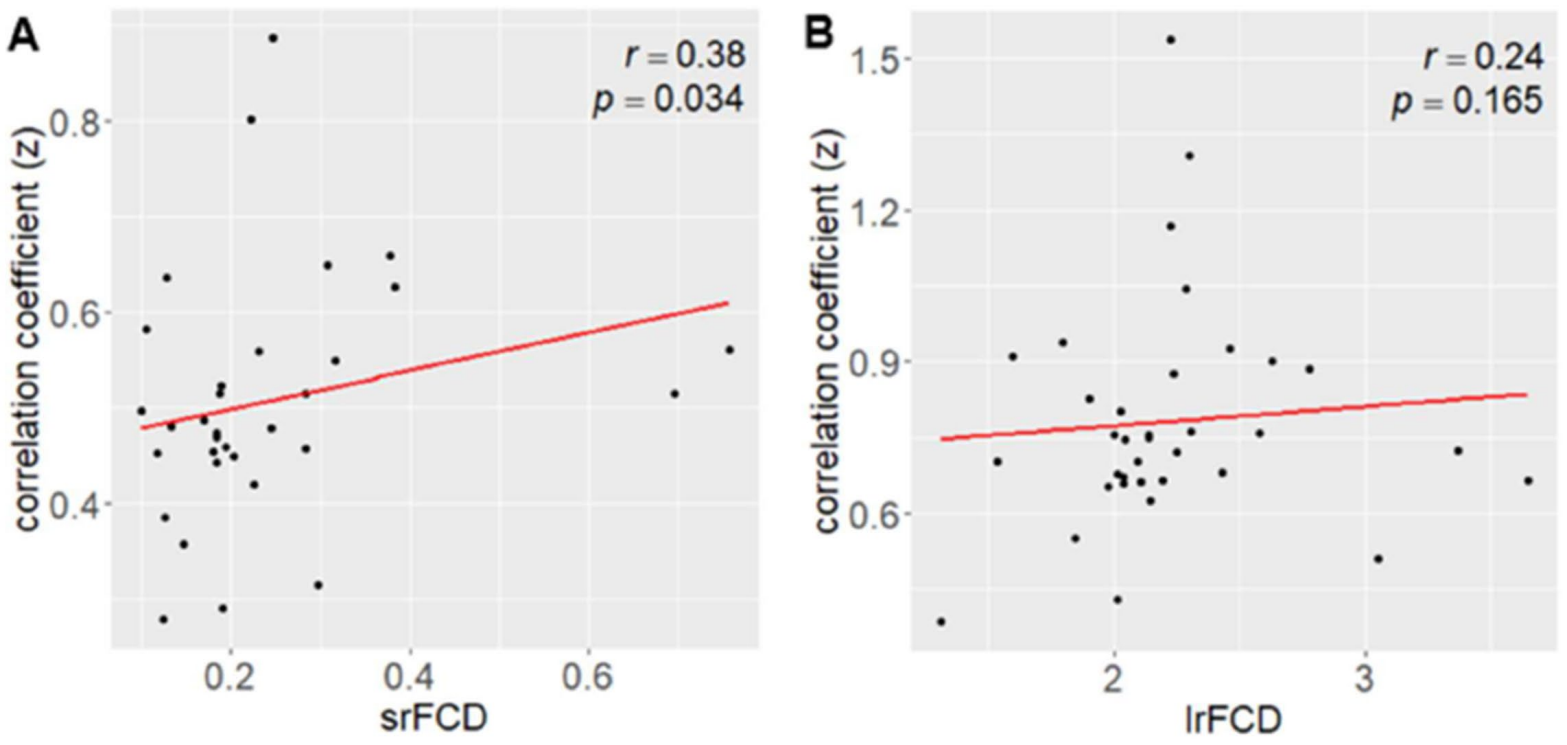
Correlation between FCD and the mean correlation coefficient between AMPAR density (SUVR) and FCD. (A) Correlation between srFCD and the mean correlation coefficient (*z* value) across the 32 areas (corresponding to Figure 2A). (B) Correlation between lrFCD and the mean correlation coefficient (*z* value) across the 41 areas (corresponding to Figure 2B). AMPAR, α-amino-3-hydroxy-5-methyl-4-isoxazole propionic acid receptor; lrFCD, long-range functional connectivity density; srFCD, short-range functional connectivity density; SUVR, standardized uptake value ratio; FCD, functional connectivity density.

We next investigated whether the absolute values of FCD were associated with the strength of the correlation between SUVR and FCD. Given that higher FCD would correspond to larger correlation coefficients, reflecting a closer relationship between SUVR and FCD in regions with high FCD. The relationship between srFCD values and the correlation coefficients between SUVR and srFCD was significantly positive (*r* = 0.38, *p* = 0.034) (Figure 4A). In contrast, lrFCD values showed no significant correlation with the correlation coefficients between SUVR and lrFCD (*r* = 0.24, *p* = 0.13) (Figure 4B). These findings suggest that intraregional functional centrality (srFCD) is closely linked to the degree of its correlation with SUVR, whereas interregional functional centrality (lrFCD) is not. However, almost all ROIs included in the DMN and VN, which showed the highest correlation coefficients between SUVR and both srFCD and lrFCD, showed relatively high srFCD, and ten of them (the bilateral cuneus: parts of the VN, the bilateral superior parietal gyrus, bilateral PCC, right angular gyrus, bilateral lateral remainder occipital lobe, and (a part of) right ACC: parts of the DMN) were included in the top ten srFCD group (Supplementary Table S1). In addition, nine regions per top ten lrFCD values group (the bilateral cuneus and right lingual gyrus: parts of the VN, the bilateral superior parietal gyrus, left PCC, right angular gyrus, left lateral remainder occipital lobe, and (a part of) left ACC: parts of the DMN) were also included in the DMN and VN (Supplementary Table S2).

### Voxel-wise regional analysis within functional networks

**Figure 5:**
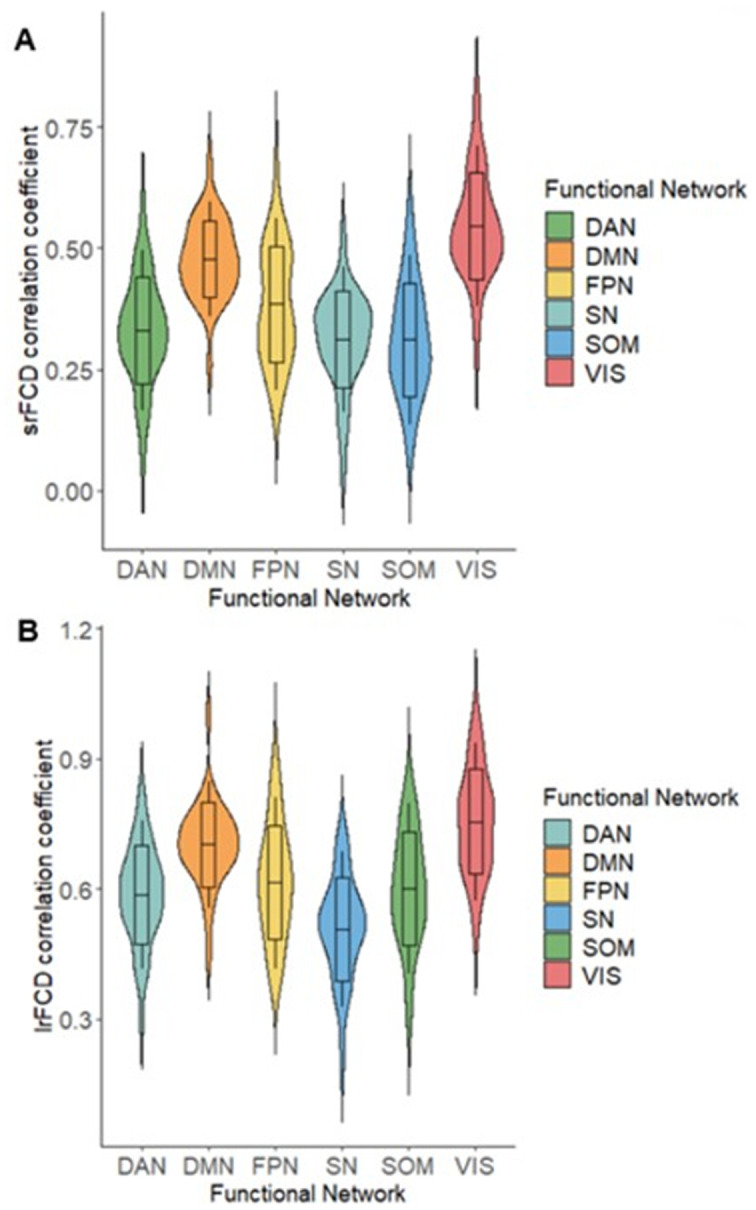
Violin plots of correlation coefficients (*z* value) between AMPAR density (SUVR) and FCD. (A) Correlation between srFCD and SUVR in 6 Networks of Yeo’s atlas. (B) Correlation between lrFCD and SUVR in 6 Networks of Yeo’s atlas. CEN: Central Executive Network, DAN: Dorsal Attention Network, DMN: Default Mode Network, SN: Salience Ventral Attention Network, SOM: Somatomotor Network, VIS: Visual Network; AMPAR, α-amino-3-hydroxy-5-methyl-4-isoxazole propionic acid receptor; lrFCD, long-range functional connectivity density; srFCD, short-range functional connectivity density; SUVR, standardized uptake value ratio; FCD, functional connectivity density.

Because task-positive and task-negative functional networks are segregations of comprehensive functions (Eickhoff et al., 2007; Thomas Yeo et al., 2011), we also examined the relationship between SUVR and FCD within different cortical functional networks. Using Yeo’s atlas (Thomas Yeo et al., 2011), which comprised 7 networks covering the whole cortex, we extracted independent SUVR, srFCD, and lrFCD images for each network. We then computed voxel-wise correlations between SUVR and srFCD or lrFCD within each network. In all seven functional networks, we found positive correlations between SUVR and both srFCD and lrFCD (Table 1) (Figures 5A,B). Notably, the correlation coefficients in DMN were among the highest, with 0.44 between SUVR and srFCD, and 0.61 between SUVR and lrFCD. Similarly, the VN showed high correlation coefficients of 0.50 between SUVR and srFCD, and 0.64 between SUVR and lrFCD (Table 1). To control spatial autocorrelation, spin-based permutation correction (Alexander-Bloch et al., 2018) was performed. Using all SUVR data in 1000 times permutation around the brain sphere, we compared the correlation of the real data with correlation using artificially permutated data between SUVR and srFCD or lrFCD for each network. The association between AMPAR and srFCD or lrFCD was significant in all networks (*p* < 0.001) except for the Limbic Network (Supplementary Tables S3, S4). These results suggest that most functional networks, particularly the DMN and VN, exhibit a robust relationship between SUVR and both intraregional and interregional functional centralities at rest.

**Table 1:** Correlation coefficients between AMPAR density (SUVR) and srFCD or lrFCD in 6 networks of Yeo’s atlas.

Network	Mean correlation coefficient (between SUVR and srFCD)	Mean correlation coefficient (between SUVR and lrFCD)
CEN	0.37 ± 0.12	0.55 ± 0.13
DAN	0.32 ± 0.11	0.53 ± 0.11
DMN	0.44 ± 0.079	0.61 ± 0.097
SN	0.30 ± 0.099	0.47 ± 0.12
Somatomotor	0.30 ± 0.12	0.54 ± 0.13
Visual	0.50 ± 0.11	0.64 ± 0.12

srFCD, short-range functional connectivity density [log (lFCD)]; lrFCD, long-range functional connectivity density [log (gFCD-lFCD)]; CEN, Central Executive Network; DAN, Dorsal Attention Network; DMN, Default Mode Network; SN, Salience Ventral Attention Network; Somatomotor, Somatomotor Network; Visual, Visual Network. Values are shown as mean ± SD. The *p*-value in each correlation in all participants was multiple comparison-adjusted (*p* < 0.05/90). After multiple comparison correction, all functional networks except for Limbic Network survived in spatial autocorrelation correction (*p* < 0.05).

## Discussion

Here, we investigated the relationship between AMPAR density and intraregional/interregional functional centrality of the whole brain, anatomical regions, and functional networks in healthy participants. We found that AMPAR density was positively correlated with srFCD and lrFCD across the whole brain as well as within the corrected anatomical ROIs and functional networks. These findings provide biological context to resting-state fMRI data, revealing a consistent positive association between AMPAR density and functional centrality across the whole brain, with regional differences observed in specific regions and networks.

### Positive correlation between AMPAR density and functional centrality in the whole brain

Across the whole brain, both srFCD and lrFCD were positively correlated with AMPAR density, suggesting that AMPAR density plays a significant role in proximal and distal neuronal communication. This leads to various cognitive processes mediated by excitatory signals in the entire brain. In an animal study, learning-induced AMPAR trafficking, increasing the amplitude of evoked synaptic transmission (Whitlock et al., 2006). In another report, injection of AMPAR agonist into the ventral tegmental area in mice augmented functional connectivities between the ventral tegmental area and the core or shell of the nucleus accumbens, as measured using fMRI (Jaime et al., 2019), which is also consistent with our study. Additionally, it was noted that administration of perampanel, an AMPAR antagonist, induced a selective increase in functional connectivities with Magnetoencephalography (MEG) in humans in the alpha band (connectivities originating from the left superior parietal lobule) and beta band (connectivities originating from the left postcentral gyrus, right inferior parietal gyrus, and left caudate) (Routley et al., 2017). The results provide collateral evidence that AMPAR density influences functional connectivity based on neural activity also in humans, which is in line with the results of our study. One study also stated that AMPAR distribution derived from autoradiography data was one of the most dominantly related neurotransmitters to MEG power as an index of functional signal, though participants who provided AMPAR data were different from living participants who provided MEG data (Hansen et al., 2022). Consistent with that previous result, this study proved that AMPAR in living humans is also strongly related to functional centralities, as comprehensive indexes of functional signal, in the same participants. Our study is consistent with a previous report that BOLD signals on rsfMRI predominantly reflect synaptic activity (Logothetis et al., 2001).

### Positive correlation between AMPAR density and functional centrality in each region and each network

Regional differences in correlations were observed between AMPAR density and FCD. The regions with the strongest correlations between AMPAR density and intraregional and interregional functional centrality included the ACC, PCC, and superior parietal gyrus (precuneus) which were part of the DMN. The correlations between AMPAR and interregional functional centrality in the psFC and caudate nucleus were also strong. The weak correlation regions between AMPAR density and intraregional functional centrality included the left cerebellum and right inferior frontal gyrus. The weakest correlation region between AMPAR density and interregional functional centrality was also the left cerebellum, though every region that has a significant correlation between AMPAR density and lrFCD did not exhibit a weak correlation. Regions with stronger correlations, including the ACC, PCC, superior parietal gyrus, caudate nucleus, and psFC can ghighdeliver more efficient signaling due to the close association between AMPAR density and functional centrality.

The correlation between AMPAR density and intra-or interregional functional centrality in each functional network was positive. These strong correlations may indicate the relevance of AMPARs in determining the functional centrality of these networks during the resting state. In this study, almost all ROIs included in the DMN and VN, which showed the highest correlation coefficients between AMPAR density and both srFCD and lrFCD, showed relatively high srFCD values, including the top ten srFCD group. Consistent with these results, a significant positive correlation was observed between srFCD and the correlation coefficient between AMPAR density and srFCD across ROIs in Hammers atlas (Figure 4A). DMN and VN also included nine regions from the top ten lrFCD value group (Supplementary Table S2). Although no significant correlation was found between lrFCD and the correlation between AMPAR density and lrFCD considering anatomical regions, it is possible that the unit of functional networks rather than the unit of anatomical regions could be related to the biological functional networks associated with AMPAR. Another study showed that regions of high lFCD can act as hubs of resting functional networks (Tomasi and Volkow, 2011). The ACC and PCC were among the regions with the highest lFCD values in the previous study (Tomasi and Volkow, 2010). These observations suggest that AMPAR density is closely correlated with functional centrality in regions that serve as functional centers in the resting-state functional networks. Overall, the differences in the correlations between AMPAR density and FCD among various anatomical regions may reflect variations in their respective biological functions at rest. The correlations between AMPAR density and srFCD or lrFCD were especially high in the DMN and VN, with the mean correlation values >0.4 or 0.6, and > 0.5 or 0.6, respectively. The DMN included the ACC, PCC, and psFC among the highest correlations between AMPAR density and srFCD or lrFCD among all the regions. Since the DMN is mostly deactivated in a task-positive state (Raichle et al., 2001; Raichle, 2015; Menon, 2023), it could be assumed that the DMN may be more related to neural activities than the other networks in a task-negative state. This phenomenon could contribute to one of the strongest correlations between AMPAR density and FCD observed in the DMN. Thus, the configuration of the DMN based on AMPAR density could add biological significance to the concept of “DMN” as a resting-state network that may be activated by synchronizing with the AMPAR density function on a synaptic level. Recent studies revealed various cognitive processes among the DMN and other networks at resting-state (Smith et al., 2018; Weber et al., 2022), which was not contradictory to our findings that correlations between AMPAR density and intra-and interregional functional centrality were positive in six functional networks, including the DMN. Another report showed that connectivities measured with MEG from several areas around the DMN, that are, the left superior parietal lobule, left superior parietal lobule, and right inferior parietal gyrus to several areas increased with the administration of an AMPAR antagonist in humans (Routley et al., 2017), which may account for the robust relationship between AMPAR density and functional centrality in the DMN at rest.

Several reasons can explain the low correlations in some regions. Significant differences were observed in the connectivities of the cerebellum to the parietal region or the frontal region between the resting state and the state while performing some tasks (Katz and Knops, 2016). Connectivity between the inferior frontal gyrus or insula and the DMN increases during tasks (Elton and Gao, 2015). Further study during task performance should be warranted to test the change in the relationship between AMPAR and FCD in these areas.

The VN mainly comprises the cuneus that showed a strong correlation between AMPAR density and intraregional and interregional functional centrality of 0.50 and 0.64, respectively. First, visual stimulation at rest could drive activation of the VN, because participants were instructed not to sleep and their eyes were open. This could lead to a strong correlation between AMPAR and FCD. It has also been reported that the VN is activated at rest and during action (Smith et al., 2009; Cornblath et al., 2020). One animal study demonstrated that AMPAR blockade in the primary visual cortex reduced cell firing frequency and diminished spatial phase-selective simple cell responses while generating phase-invariant complex cell responses (Rivadulla et al., 2001). Furthermore, another previous study showed that an increase of serotonin secretion at layer 4 to layer 2/3 synapses in the barrel cortex of mice after being deprived of their vision promoted trafficking of AMPARs and then enhanced excitatory input, improving whisker tactile function (Jitsuki et al., 2011). These compensatory cortical reorganizations of synapses may support the strong correlation between AMPAR density and functional networks in vision.

We considered several reasons that the association between AMPAR and FCD was not significant in the Limbic Network. FCD, which reflects the BOLD signal, is sensitive to blood volume and blood oxygenation and is influenced by tissue-specific factors like cellular density, myelination, water content, iron content, and vascularization. These elements collectively contribute to the observed lower signal-to-noise in the Limbic Network, with vascularization playing an important role in signal variation within T2*-weighted imaging. Furthermore, MRI hardware limitations further exacerbate the lower SNR in limbic regions. Specifically, the RF coil geometry and design limit RF penetration into deeper structures, reducing SNR in areas like the hippocampus, amygdala, and related limbic regions. Additionally, B1+ inhomogeneity, which we addressed using N4 bias field correction, may further impact SNR in these areas, though current correction methods may not fully resolve this issue. Cortical regions, by comparison, show higher SNR due to their closer proximity to RF coil elements, which enhances spatial image quality. The Limbic Network is also associated with emotional processing (Dan et al., 2023), and the resting state in this study scarcely demanded any emotional processing. Further investigation is necessary for confirmation of the correlation between AMPAR density and srFCD or lrFCD in the Limbic Network in performing some tasks, especially emotional tasks. This is essential because AMPAR plays a crucial role in the basis of experience-dependent synaptic plasticity (Takahashi et al., 2003; Kessels and Malinow, 2009; Mitsushima et al., 2011; Mitsushima et al., 2013) and may be related to emotional experience-dependent fMRI signal activation.

### Limitations

This study had some limitations. First, the direction or destination of the functional connectivity was not examined. Secondly, we did not interview the state of consciousness during rsfMRI scanning in our participants after the scanning. The relationship between cognitive performance or the state of consciousness and the correlation between AMPAR density and functional centrality should be warranted in the future. Third, brain receptors other than AMPARs were not considered. Finally, although we found a strong relationship between AMPAR and the degree of functional connections in the whole brain, the impact of AMPAR density on the strength of functional connectivity was not assessed.

## Conclusion

In conclusion, this study suggests that AMPAR density plays an important role in modulating functional centrality in the resting-state brain. Since functional networks are activated during tasks, task-based fMRI studies are warranted to understand the dynamic interaction between AMPAR and functional centrality. Moreover, comparisons between healthy individuals and patients with psychiatric disorders may deepen our understanding of the pathophysiological mechanisms underlying these disorders. For example, using the correlation between SUVR and srFCD or lrFCD revealed with this study, comparison between healthy subjects and psychiatric disorders including schizophrenia, depression, bipolar disorders, and autism spectrum disorders in the whole brain, in functional networks, and in some anatomical brain resions should be investigated. The findings of this study could help develop strategies benefiting the studies of the brain’s functions and brain diseases.

## Data Availability

The data analyzed in this study is subject to the following licenses/restrictions: all requests for raw and analyzed data and codes used in this study are promptly reviewed by the Yokohama City University Research Promotion Department to determine whether the request is subject to any intellectual property or confidentiality obligations and further inspected by the Institutional Review Board of Yokohama City University Hospital. Upon these approvals, derived data will be released via a material transfer agreement from the corresponding author. Requests to access these datasets should be directed to Takuya Takahashi, takahast@yokohama-cu.ac.jp.

## Footnotes

^1^http://hdl.handle.net/10380/3113

^2^https://surfer.nmr.mgh.harvard.edu/
