# Corrigendum: α-amino-3-hydroxy-5-methyl-4-isoxazole propionic acid (AMPA) receptor density underlies intraregional and interregional functional centrality

**DOI:** 10.3389/fncir.2024.1533008

**Published:** 2024-12-10

**Authors:** Taisuke Yatomi, Dardo Tomasi, Hideaki Tani, Shinichiro Nakajima, Sakiko Tsugawa, Nobuhiro Nagai, Teruki Koizumi, Waki Nakajima, Mai Hatano, Hiroyuki Uchida, Takuya Takahashi

**Affiliations:** ^1^Department of Neuropsychiatry, Keio University School of Medicine, Tokyo, Japan; ^2^Department of Physiology, Yokohama City University Graduate School of Medicine, Yokohama, Japan; ^3^Laboratory of Neuroimaging (LNI), National Institute on Alcohol Abuse and Alcoholism, NIH, Bethesda, MD, United States

**Keywords:** α-amino-3-hydroxy-5-methyl-4-isoxazole propionic acid (AMPA) receptor, [^11^C]K-2, positron emission tomography, synaptic plasticity, resting-state functional MRI (rsfMRI), functional connectivity density mapping, functional network, functional centrality

In the published article, there was an error in the order of affiliation 2 and 3. Instead of “^2^Laboratory of Neuroimaging (LNI), National Institute on Alcohol Abuse and Alcoholism, NIH, Bethesda, MD, United States, ^3^Department of Physiology, Yokohama City University Graduate School of Medicine, Yokohama, Japan”, affiliation 2 and 3 should have been correctly written as “^2^Department of Physiology, Yokohama City University Graduate School of Medicine, Yokohama, Japan, ^3^Laboratory of Neuroimaging (LNI), National Institute on Alcohol Abuse and Alcoholism, NIH, Bethesda, MD, United States”.

The authors apologize for this error and state that this does not change the scientific conclusions of the article in any way. The original article has been updated.

