# Supplementary figures and images for: α-amino-3-hydroxy-5-methyl-4-isoxazole propionic acid (AMPA) receptor density underlies intraregional and interregional functional centrality
Source: Front Neural Circuits. 2024 Nov 6;18:1497897. doi: 10.3389/fncir.2024.1497897 (PMC11576226; doi:10.3389/fncir.2024.1497897)

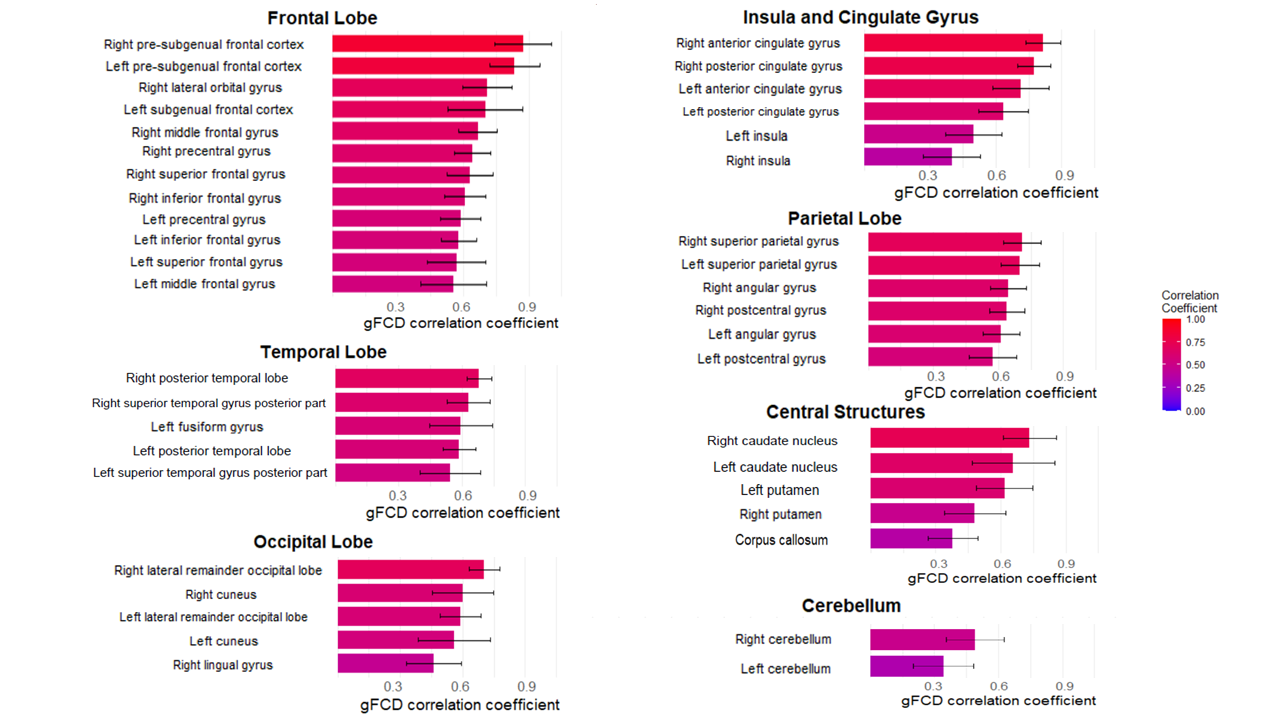

Supplement: Supplementary file 1 [file Image_1.TIF]
