# Supplementary material for: α-amino-3-hydroxy-5-methyl-4-isoxazole propionic acid (AMPA) receptor density underlies intraregional and interregional functional centrality
Source: Front Neural Circuits. 2024 Nov 6;18:1497897. doi: 10.3389/fncir.2024.1497897 (PMC11576226; doi:10.3389/fncir.2024.1497897)
Supplement: Supplementary file 2 [file Data_Sheet_1.docx]

Supplementary Material

# Supplementary Table Legends

**Table S1**. The mean values of srFCD in each ROI in Hammers atlas. Only multiple comparison correction-survived 36 regions (corresponding to Figure 2A) were displayed. Values are shown as mean ± SD.

**Table S2**. The mean values of lrFCD in each ROI in Hammers atlas. Only multiple comparison correction-survived 46 regions (corresponding to Figure 2B) were displayed. Values are shown as mean ± SD.

**Table S3**. Results of the spin-test in the correlation between SUVR and srFCD about the survived 33 regions and 7 networks in step 1. Each p-value is the one that is the number of the null correlations outperforming the real correlation being divided by 1000 in each ROI or network. p < 0.001 means that the real correlation denied the null hypothesis all times. CEN: Central Executive Network, DAN: Dorsal Attention Network, DMN: Default Mode Network, LIM: Limbic Network, SN: Salience Ventral Attention Network, SOM: Somatomotor Network, VIS: Visual Network.

**Table S4**. Results of the spin-test in the correlation between SUVR and lrFCD about the survived 40 regions and 7 networks in step 1. Each p-value is the one that is the number of the null correlations outperforming the real correlation being divided by 1000 in each ROI or network. p < 0.001 means that the real correlation denied the null hypothesis all times. CEN: Central Executive Network, DAN: Dorsal Attention Network, DMN: Default Mode Network, LIM: Limbic Network, SN: Salience Ventral Attention Network, SOM: Somatomotor Network, VIS: Visual Network.

**Table S5**. Results of the spin-test in the correlation between SUVR and gFCD about the survived 40 regions and 7 networks in step 1. Each p-value is the one that is the number of the null correlations outperforming the real correlation being divided by 1000 in each ROI or network. p < 0.001 means that the real correlation denied the null hypothesis all times. CEN: Central Executive Network, DAN: Dorsal Attention Network, DMN: Default Mode Network, LIM: Limbic Network, SN: Salience Ventral Attention Network, SOM: Somatomotor Network, VIS: Visual Network.

**Table S6**. The results of the Kolmogorov-Smirnov test in the SUVR, srFCD, lrFCD, and gFCD images in thirty-five healthy participants (shown as HC01 to HC35 in the Table). D: statistical value of the Kolmogorov-Smirnov test, p-value: p-value against the null hypothesis about the normality distribution. P-value < 0.05 was defined as significant.

**Table S7.** The results of the Shapiro-Wilk test of the mean correlations between SUVR and srFCD, srFCD mean values in 83 ROIs in Hammers Atlas in thirty-five healthy participants, correlations between SUVR and lrFCD, and lrFCD mean values in 83 ROIs in Hammers Atlas. W: statistical value of the Shapiro-Wilk test, p-value: p-value for testing the null hypothesis that each variable follows a normal distribution. P-value < 0.05 was defined as significant.

# Supplementary Figure Legend

**Figure S1.** The mean correlation coefficients of multiple comparison-corrected and spatial autocorrelation-corrected 41 areas in Hammers atlas between AMPAR density (SUVR) and gFCD in 35 healthy participants. The bar graphs in the right column shows the mean correlation coefficient between SUVR and gFCD. The bar graphs in the right column shows the mean correlation coefficient between SUVR and lrFCD. The names in the center at the same height as these bars are those of the corresponding brain regions in Hammers Atlas. The large headings follow the major divisions of Hammers Atlas. Bars represent the mean and whiskers show the SD.

**Table S1**. The mean values of srFCD in each ROI in Hammers atlas. Only multiple comparison correction-survived 36 regions (corresponding to Figure 2A) were displayed. Values are shown as mean ± SD.

| ROI | srFCD |
| --- | --- |
| Left cuneus | 0.76 ± 0.42 |
| Right cuneus | 0.70 ± 0.41 |
| Left superior parietal gyrus | 0.38 ± 0.10 |
| Right superior parietal gyrus | 0.38 ± 0.11 |
| Left posterior cingulate gyrus | 0.32 ± 0.12 |
| Right posterior cingulate gyrus | 0.31 ± 0.13 |
| Right angular gyrus | 0.30 ± 0.11 |
| Left lateral remainder occipital lobe | 0.28 ± 0.12 |
| Right lateral remainder occipital lobe | 0.28 ± 0.12 |
| Right anterior cingulate gyrus | 0.25 ± 0.081 |
| Left angular gyrus | 0.24 ± 0.074 |
| Right superior frontal gyrus | 0.23 ± 0.050 |
| Right postcentral gyrus | 0.23 ± 0.067 |
| Left anterior cingulate gyrus | 0.22 ± 0.062 |
| Left postcentral gyrus | 0.20 ± 0.064 |
| Right precentral gyrus | 0.19 ± 0.052 |
| Left posterior temporal lobe | 0.19 ± 0.055 |
| Right inferior frontal gyrus | 0.19 ± 0.051 |
| Left middle frontal gyrus | 0.19 ± 0.052 |
| Right posterior temporal lobe | 0.19 ± 0.061 |
| Left precentral gyrus | 0.18 ± 0.051 |
| Right middle frontal gyrus | 0.18 ± 0.054 |
| Right superior temporal gyrus posterior part | 0.18 ± 0.061 |
| Left inferior frontal gyrus | 0.18 ± 0.055 |
| Left superior temporal gyrus posterior part | 0.17 ± 0.052 |
| Right output insula | 0.15 ± 0.034 |
| Left output insula | 0.13 ± 0.027 |
| Right lateral orbital gyrus | 0.13 ± 0.040 |
| Right putamen | 0.13 ± 0.029 |
| Left cerebellum | 0.12 ± 0.032 |
| Right cerebellum | 0.12 ± 0.034 |
| Right parahippocampal and ambient gyrus | 0.11 ± 0.037 |
| Right medial orbital gyrus | 0.11 ± 0.066 |
| Left fusiform gyrus | 0.10 ± 0.035 |
| Right anterior orbital gyrus | 0.099 ± 0.054 |
| Right middle and inferior temporal gyrus | 0.072 ± 0.029 |

**Table S2**. The mean values of lrFCD in each ROI in Hammers atlas. Only multiple comparison correction-survived 46 regions (corresponding to Figure 2B) were displayed. Values are shown as mean ± SD.

| ROI | lrFCD |
| --- | --- |
| Left cuneus | 3.6 ± 0.89 |
| Right cuneus | 3.4 ± 0.80 |
| Right lingual gyrus | 3.1 ± 0.70 |
| Left superior parietal gyrus | 2.8 ± 0.29 |
| Right superior parietal gyrus | 2.6 ± 0.33 |
| Left posterior cingulate gyrus | 2.6 ± 0.39 |
| Left anterior cingulate gyrus | 2.5 ± 0.32 |
| Left lateral remainder occipital lobe | 2.4 ± 0.47 |
| Right angular gyrus | 2.3 ± 0.33 |
| Left pre-subgenual frontal cortex | 2.3 ± 0.45 |
| Right posterior cingulate gyrus | 2.3 ± 0.35 |
| Left angular gyrus | 2.3 ± 0.27 |
| Right lateral remainder occipital lobe | 2.2 ± 0.40 |
| Right anterior cingulate gyrus | 2.2 ± 0.29 |
| Right pre-subgenual frontal cortex | 2.2 ± 0.53 |
| Left superior frontal gyrus | 2.2 ± 0.18 |
| Left superior temporal gyrus posterior part | 2.1 ± 0.30 |
| Right postcentral gyrus | 2.1 ± 0.27 |
| Right superior frontal gyrus | 2.1 ± 0.23 |
| Left postcentral gyrus | 2.1 ± 0.26 |
| Right inferior frontal gyrus | 2.1 ± 0.26 |
| Right superior temporal gyrus posterior part | 2.0 ± 0.27 |
| Left posterior temporal lobe | 2.0 ± 0.23 |
| Left inferior frontal gyrus | 2.0 ± 0.21 |
| Right middle frontal gyrus | 2.0 ± 0.22 |
| Left precentral gyrus | 2.0 ± 0.24 |
| Right output insula | 2.0 ± 0.25 |
| Right precentral gyrus | 2.0 ± 0.22 |
| Left middle frontal gyrus | 2.0 ± 0.19 |
| Right posterior temporal lobe | 1.9 ± 0.26 |
| Right superior temporal gyrus anterior part | 1.9 ± 0.30 |
| Left output insula | 1.8 ± 0.21 |
| Right parahippocampal and ambient gyrus | 1.8 ± 0.40 |
| Left subgenual frontal cortex | 1.8 ± 0.29 |
| Right putamen | 1.8 ± 0.21 |
| Left putamen | 1.7 ± 0.18 |
| Left cerebellum | 1.7 ± 0.26 |
| Right lateral orbital gyrus | 1.6 ± 0.36 |
| Left fusiform gyrus | 1.5 ± 0.32 |
| Right cerebellum | 1.5 ± 0.26 |
| Left caudate nucleus | 1.5 ± 0.20 |
| Right caudate nucleus | 1.5 ± 0.18 |
| Left lateral orbital gyrus | 1.4 ± 0.36 |
| corpus callosum | 1.3 ± 0.20 |
| Right medial orbital gyrus | 1.3 ± 0.36 |
| Right middle and inferior temporal gyrus | 1.0 ± 0.28 |

**Table S3**. Results of the spin-test in the correlation between SUVR and srFCD about the survived 33 regions and 7 networks in step 1. Each p-value is the one that is the number of the null correlations outperforming the real correlation being divided by 1000 in each ROI or network. p < 0.001 means that the real correlation denied the null hypothesis all times. CEN: Central Executive Network, DAN: Dorsal Attention Network, DMN: Default Mode Network, LIM: Limbic Network, SN: Salience Ventral Attention Network, SOM: Somatomotor Network, VIS: Visual Network.

| ROI | p-value | Correlation coefficient  of spin-test |
| --- | --- | --- |
| Right anterior cingulate gyrus | 0.001 | 0.79 |
| Left anterior cingulate gyrus | 0.001 | 0.82 |
| Right superior parietal gyrus | < 0.001 | 0.80 |
| Right posterior cingulate gyrus | 0.006 | 0.63 |
| Right lateral orbital gyrus | 0.009 | 0.68 |
| Left superior parietal gyrus | < 0.001 | 0.70 |
| Right medial orbital gyrus | 0.049 | 0.63 |
| Left cuneus | 0.003 | 0.75 |
| Right superior frontal gyrus | < 0.001 | 0.64 |
| Left posterior cingulate gyrus | 0.008 | 0.55 |
| Left middle frontal gyrus | < 0.001 | 0.68 |
| Right cuneus | 0.007 | 0.73 |
| Right posterior temporal lobe | < 0.001 | 0.58 |
| Right lateral remainder occipital lobe | < 0.001 | 0.56 |
| Right anterior orbital gyrus | 0.014 | 0.58 |
| Left superior temporal gyrus posterior part | < 0.001 | 0.71 |
| Left insula | 0.007 | 0.53 |
| Left angular gyrus | 0.002 | 0.50 |
| Right middle frontal gyrus | < 0.001 | 0.73 |
| Left precentral gyrus | < 0.001 | 0.55 |
| Right precentral gyrus | 0.001 | 0.51 |
| Left lateral remainder occipital lobe | 0.004 | 0.40 |
| Left inferior frontal gyrus | 0.001 | 0.74 |
| Left postcentral gyrus | < 0.001 | 0.68 |
| Left superior temporal gyrus posterior part | 0.003 | 0.68 |
| Right postcentral gyrus | < 0.001 | 0.67 |
| Right insula | 0.038 | 0.47 |
| Right angular gyrus | < 0.001 | 0.65 |
| Right inferior frontal gyrus | 0.001 | 0.83 |
| Left posterior temporal lobe | 0.22 | 0.15 |
| Left fusiform gyrus | 0.069 | 0.45 |
| Right middle and inferior temporal gyrus | 0.73 | 0.089 |
| Right parahippocampal and ambient gyrus | 0.521 | 0.180836 |
|  |  |  |
| Network |  |  |
| VIS | < 0.001 | 0.69 |
| SOM | < 0.001 | 0.53 |
| DAN | < 0.001 | 0.58 |
| SN | < 0.001 | 0.54 |
| LIM | 0.31 | 0.11 |
| FPN | < 0.001 | 0.55 |
| DMN | < 0.001 | 0.58 |

**Table S4**. Results of the spin-test in the correlation between SUVR and lrFCD about the survived 40 regions and 7 networks in step 1. Each p-value is the one that is the number of the null correlations outperforming the real correlation being divided by 1000 in each ROI or network. p < 0.001 means that the real correlation denied the null hypothesis all times. CEN: Central Executive Network, DAN: Dorsal Attention Network, DMN: Default Mode Network, LIM: Limbic Network, SN: Salience Ventral Attention Network, SOM: Somatomotor Network, VIS: Visual Network.

| ROI | p-value | Correlation coefficient  of spin-test |
| --- | --- | --- |
| Right pre-subgenual frontal cortex | 0.003 | 0.97 |
| Left pre-subgenual frontal cortex | 0.004 | 0.99 |
| Right anterior cingulate gyrus | 0.001 | 0.87 |
| Right posterior cingulate gyrus | 0.002 | 0.87 |
| Left anterior cingulate gyrus | 0.001 | 0.80 |
| Right superior parietal gyrus | < 0.001 | 0.87 |
| Right lateral orbital gyrus | 0.013 | 0.70 |
| Right lateral remainder occipital lobe | < 0.001 | 0.77 |
| Left subgenual frontal cortex | 0.002 | 0.91 |
| Left superior parietal gyrus | < 0.001 | 0.84 |
| Right posterior temporal lobe | < 0.001 | 0.66 |
| Right middle frontal gyrus | < 0.001 | 0.85 |
| Right angular gyrus | < 0.001 | 0.78 |
| Right precentral gyrus | < 0.001 | 0.79 |
| Right postcentral gyrus | < 0.001 | 0.83 |
| Left posterior cingulate gyrus | < 0.001 | 0.83 |
| Right superior temporal gyrus posterior part | < 0.001 | 0.83 |
| Right superior frontal gyrus | < 0.001 | 0.82 |
| Left angular gyrus | < 0.001 | 0.67 |
| Right inferior frontal gyrus | < 0.001 | 0.91 |
| Right cuneus | 0.003 | 0.86 |
| Left fusiform gyrus | 0.032 | 0.54 |
| Left lateral remainder occipital lobe | < 0.001 | 0.69 |
| Left precentral gyrus | < 0.001 | 0.72 |
| Left posterior temporal lobe | < 0.001 | 0.47 |
| Left inferior frontal gyrus | < 0.001 | 0.81 |
| Left postcentral gyrus | < 0.001 | 0.75 |
| Left superior frontal gyrus | < 0.001 | 0.57 |
| Left cuneus | 0.001 | 0.86 |
| Left middle frontal gyrus | < 0.001 | 0.64 |
| Left superior temporal gyrus posterior part | 0.002 | 0.67 |
| Left insula | 0.005 | 0.61 |
| Right lingual gyrus | 0.003 | 0.85 |
| Right insula | 0.013 | 0.57 |
| Corpus callosum | 0.003 | 0.67 |
| Right medial orbital gyrus | 0.12 | 0.41 |
| Left lateral orbital gyrus | 0.51 | 0.31 |
| Right middle and inferior temporal gyrus | 0.80 | -0.067 |
| Left parahippocampal and ambient gyrus | 0.95 | 0.020 |
| Right superior temporal gyrus anterior part | 0.32 | 0.29 |
|  |  |  |
| Network |  |  |
| VIS | < 0.001 | 0.80 |
| SOM | < 0.001 | 0.74 |
| DAN | < 0.001 | 0.73 |
| SN | < 0.001 | 0.72 |
| LIM | 0.84 | 0.026 |
| FPN | < 0.001 | 0.70 |
| DMN | < 0.001 | 0.63 |

**Table S5**. Results of the spin-test in the correlation between SUVR and gFCD about the survived 40 regions and 7 networks in step 1. Each p-value is the one that is the number of the null correlations outperforming the real correlation being divided by 1000 in each ROI or network. p < 0.001 means that the real correlation denied the null hypothesis all times. CEN: Central Executive Network, DAN: Dorsal Attention Network, DMN: Default Mode Network, LIM: Limbic Network, SN: Salience Ventral Attention Network, SOM: Somatomotor Network, VIS: Visual Network.

| ROI | p-value | Correlation coefficient  of spin-test |
| --- | --- | --- |
| Right pre-subgenual frontal cortex | 0.003 | 0.97 |
| Left pre-subgenual frontal cortex | 0.004 | 0.99 |
| Right anterior cingulate gyrus | 0.001 | 0.86 |
| Right posterior cingulate gyrus | 0.002 | 0.91 |
| Right superior parietal gyrus | < 0.001 | 0.87 |
| Left anterior cingulate gyrus | 0.001 | 0.79 |
| Right lateral orbital gyrus | 0.015 | 0.71 |
| Left superior parietal gyrus | < 0.001 | 0.85 |
| Right lateral remainder occipital lobe | < 0.001 | 0.78 |
| Left subgenual frontal cortex | 0.003 | 0.98 |
| Right posterior temporal lobe | < 0.001 | 0.83 |
| Right middle frontal gyrus | < 0.001 | 0.85 |
| Right angular gyrus | < 0.001 | 0.78 |
| Right postcentral gyrus | < 0.001 | 0.88 |
| Right precentral gyrus | < 0.001 | 0.84 |
| Left posterior cingulate gyrus | < 0.001 | 0.95 |
| Right superior frontal gyrus | < 0.001 | 0.82 |
| Right superior temporal gyrus posterior part | < 0.001 | 0.89 |
| Left angular gyrus | < 0.001 | 0.76 |
| Right cuneus | 0.003 | 0.86 |
| Right inferior frontal gyrus | < 0.001 | 0.91 |
| Left fusiform gyrus | < 0.001 | 0.91 |
| Left precentral gyrus | < 0.001 | 0.85 |
| Left lateral remainder occipital lobe | < 0.001 | 0.75 |
| Left posterior temporal lobe | < 0.001 | 0.81 |
| Left inferior frontal gyrus | < 0.001 | 0.92 |
| Left postcentral gyrus | < 0.001 | 0.90 |
| Left superior frontal gyrus | < 0.001 | 0.68 |
| Left middle frontal gyrus | < 0.001 | 0.79 |
| Left cuneus | 0.001 | 0.86 |
| Left superior temporal gyrus posterior part | < 0.001 | 0.83 |
| Left insula | 0.004 | 0.66 |
| Right lingual gyrus | 0.003 | 0.84 |
| Right insula | 0.013 | 0.57 |
| corpus callosum | < 0.001 | 0.99 |
| Right medial orbital gyrus | 0.077 | 0.49 |
| Left lateral orbital gyrus | 0.48 | 0.33 |
| Right middle and inferior temporal gyrus | 0.54 | 0.15 |
| Right parahippocampal and ambient gyrus | 0.53 | 0.15 |
| Left superior temporal gyrus anterior part | 0.89 | 0.036 |
|  |  |  |
| Network |  |  |
| VIS | < 0.001 | 0.80 |
| SOM | < 0.001 | 0.80 |
| DAN | < 0.001 | 0.75 |
| SN | < 0.001 | 0.85 |
| LIM | 0.51 | 0.080 |
| FPN | < 0.001 | 0.76 |
| DMN | < 0.001 | 0.66 |

**Table S6**. The results of the Kolmogorov-Smirnov test in the SUVR, srFCD, lrFCD, and gFCD images in thirty-five healthy participants (shown as HC01 to HC35 in the Table). D: statistical value of the Kolmogorov-Smirnov test, p-value: p-value against the null hypothesis about the normality distribution. P-value < 0.05 was defined as significant.

|  | SUVR |  | srFCD |  | lrFCD |  | gFCD |  |
| --- | --- | --- | --- | --- | --- | --- | --- | --- |
| ID | D | p-value | D | p-value | D | p-value | D | p-value |
| HC01 | 0.84 | < 2.2×10^-16^ | 0.50 | < 2.2×10^-16^ | 0.74 | < 2.2×10^-16^ | 0.76 | < 2.2×10^-16^ |
| HC02 | 0.88 | < 2.2×10^-16^ | 0.50 | < 2.2×10^-16^ | 0.71 | < 2.2×10^-16^ | 0.72 | < 2.2×10^-16^ |
| HC03 | 0.87 | < 2.2×10^-16^ | 0.50 | < 2.2×10^-16^ | 0.68 | < 2.2×10^-16^ | 0.69 | < 2.2×10^-16^ |
| HC04 | 0.87 | < 2.2×10^-16^ | 0.50 | < 2.2×10^-16^ | 0.74 | < 2.2×10^-16^ | 0.75 | < 2.2×10^-16^ |
| HC05 | 0.87 | < 2.2×10^-16^ | 0.50 | < 2.2×10^-16^ | 0.73 | < 2.2×10^-16^ | 0.74 | < 2.2×10^-16^ |
| HC06 | 0.84 | < 2.2×10^-16^ | 0.50 | < 2.2×10^-16^ | 0.76 | < 2.2×10^-16^ | 0.77 | < 2.2×10^-16^ |
| HC07 | 0.86 | < 2.2×10^-16^ | 0.50 | < 2.2×10^-16^ | 0.72 | < 2.2×10^-16^ | 0.73 | < 2.2×10^-16^ |
| HC08 | 0.87 | < 2.2×10^-16^ | 0.50 | < 2.2×10^-16^ | 0.67 | < 2.2×10^-16^ | 0.69 | < 2.2×10^-16^ |
| HC09 | 0.87 | < 2.2×10^-16^ | 0.50 | < 2.2×10^-16^ | 0.77 | < 2.2×10^-16^ | 0.78 | < 2.2×10^-16^ |
| HC10 | 0.86 | < 2.2×10^-16^ | 0.50 | < 2.2×10^-16^ | 0.72 | < 2.2×10^-16^ | 0.73 | < 2.2×10^-16^ |
| HC11 | 0.86 | < 2.2×10^-16^ | 0.50 | < 2.2×10^-16^ | 0.70 | < 2.2×10^-16^ | 0.71 | < 2.2×10^-16^ |
| HC12 | 0.87 | < 2.2×10^-16^ | 0.50 | < 2.2×10^-16^ | 0.64 | < 2.2×10^-16^ | 0.66 | < 2.2×10^-16^ |
| HC13 | 0.86 | < 2.2×10^-16^ | 0.50 | < 2.2×10^-16^ | 0.70 | < 2.2×10^-16^ | 0.71 | < 2.2×10^-16^ |
| HC14 | 0.88 | < 2.2×10^-16^ | 0.50 | < 2.2×10^-16^ | 0.72 | < 2.2×10^-16^ | 0.73 | < 2.2×10^-16^ |
| HC15 | 0.87 | < 2.2×10^-16^ | 0.50 | < 2.2×10^-16^ | 0.73 | < 2.2×10^-16^ | 0.74 | < 2.2×10^-16^ |
| HC16 | 0.88 | < 2.2×10^-16^ | 0.50 | < 2.2×10^-16^ | 0.74 | < 2.2×10^-16^ | 0.75 | < 2.2×10^-16^ |
| HC17 | 0.86 | < 2.2×10^-16^ | 0.50 | < 2.2×10^-16^ | 0.69 | < 2.2×10^-16^ | 0.70 | < 2.2×10^-16^ |
| HC18 | 0.85 | < 2.2×10^-16^ | 0.50 | < 2.2×10^-16^ | 0.74 | < 2.2×10^-16^ | 0.75 | < 2.2×10^-16^ |
| HC19 | 0.86 | < 2.2×10^-16^ | 0.50 | < 2.2×10^-16^ | 0.65 | < 2.2×10^-16^ | 0.66 | < 2.2×10^-16^ |
| HC20 | 0.86 | < 2.2×10^-16^ | 0.50 | < 2.2×10^-16^ | 0.72 | < 2.2×10^-16^ | 0.73 | < 2.2×10^-16^ |
| HC21 | 0.85 | < 2.2×10^-16^ | 0.50 | < 2.2×10^-16^ | 0.77 | < 2.2×10^-16^ | 0.78 | < 2.2×10^-16^ |
| HC22 | 0.87 | < 2.2×10^-16^ | 0.50 | < 2.2×10^-16^ | 0.78 | < 2.2×10^-16^ | 0.79 | < 2.2×10^-16^ |
| HC23 | 0.86 | < 2.2×10^-16^ | 0.50 | < 2.2×10^-16^ | 0.74 | < 2.2×10^-16^ | 0.75 | < 2.2×10^-16^ |
| HC24 | 0.85 | < 2.2×10^-16^ | 0.50 | < 2.2×10^-16^ | 0.73 | < 2.2×10^-16^ | 0.74 | < 2.2×10^-16^ |
| HC25 | 0.86 | < 2.2×10^-16^ | 0.50 | < 2.2×10^-16^ | 0.72 | < 2.2×10^-16^ | 0.73 | < 2.2×10^-16^ |
| HC26 | 0.87 | < 2.2×10^-16^ | 0.50 | < 2.2×10^-16^ | 0.72 | < 2.2×10^-16^ | 0.74 | < 2.2×10^-16^ |
| HC27 | 0.86 | < 2.2×10^-16^ | 0.50 | < 2.2×10^-16^ | 0.72 | < 2.2×10^-16^ | 0.73 | < 2.2×10^-16^ |
| HC28 | 0.85 | < 2.2×10^-16^ | 0.50 | < 2.2×10^-16^ | 0.72 | < 2.2×10^-16^ | 0.73 | < 2.2×10^-16^ |
| HC29 | 0.86 | < 2.2×10^-16^ | 0.50 | < 2.2×10^-16^ | 0.73 | < 2.2×10^-16^ | 0.74 | < 2.2×10^-16^ |
| HC30 | 0.87 | < 2.2×10^-16^ | 0.50 | < 2.2×10^-16^ | 0.73 | < 2.2×10^-16^ | 0.74 | < 2.2×10^-16^ |
| HC31 | 0.86 | < 2.2×10^-16^ | 0.50 | < 2.2×10^-16^ | 0.72 | < 2.2×10^-16^ | 0.73 | < 2.2×10^-16^ |
| HC32 | 0.85 | < 2.2×10^-16^ | 0.50 | < 2.2×10^-16^ | 0.73 | < 2.2×10^-16^ | 0.75 | < 2.2×10^-16^ |
| HC33 | 0.85 | < 2.2×10^-16^ | 0.50 | < 2.2×10^-16^ | 0.80 | < 2.2×10^-16^ | 0.80 | < 2.2×10^-16^ |
| HC34 | 0.85 | < 2.2×10^-16^ | 0.50 | < 2.2×10^-16^ | 0.73 | < 2.2×10^-16^ | 0.74 | < 2.2×10^-16^ |
| HC35 | 0.87 | < 2.2×10^-16^ | 0.50 | < 2.2×10^-16^ | 0.72 | < 2.2×10^-16^ | 0.74 | < 2.2×10^-16^ |

**Table S7.** The results of the Shapiro-Wilk test of correlations between SUVR and srFCD, srFCD mean values in 83 Hammers Atlas ROIs, correlations between SUVR and lrFCD, and lrFCD mean values in 83 Hammers Atlas ROIs. W: statistical value of the Shapiro-Wilk test, p-value: p-value against the null hypothesis of the normal distribution in each values. P-value < 0.05 was defined as significant.

|  | W | p-value |
| --- | --- | --- |
| srFCD correlation coefficients (z) | 0.94 | 0.055 |
| srFCD values | 0.73 | 2.5×10^-6^ |
| lrFCD correlation coefficients (z) | 0.91 | 0.0048 |
| lrFCD values | 0.92 | 0.0074 |
